# Falls requiring visit to emergency room in a population-based cohort of diabetic patients in Italy

**DOI:** 10.5249/jivr.v9i2.859

**Published:** 2017-07

**Authors:** Francesca Valent

**Affiliations:** ^*a*^Servizio Epidemiologico – Direzione Centrale Salute, Integrazione Sociosanitaria, Politiche Sociali e Famiglia – Regione Autonoma Friuli Venezia Giulia – Udine, Italy.

**Keywords:** Diabetes mellitus, Accidental falls, Italy, Emergency department

## Abstract

**Background:**

The aims were to assess the frequency of falls among the diabetic adult population of the Italian Northeastern region Friuli Venezia Giulia and to identify risk factors.

**Methods:**

This was a population-based retrospective cohort study using administrative data of the regional health information system as the source of information. In a cohort of diabetics 18 years of age or more, living in the region on December 31, 2014, the occurrence of falls requiring a visit to the regional Emergency Rooms was assessed. Multivariate logistic regression was used to identify factors associated with increased risk of falling.

**Results:**

Of 80,162 cohort subjects, 2967 (3.7%) had at least one fall requiring a visit to ER. Factors associated with increased risk of falling were female sex, older age, prescription of a thiazolidinedione as the last antidiabetic medication in 2014, increasing number of active principles prescribed in 2014, longer diabetes duration, and prescription of certain classes of medications other than antidiabetics in 2014.

**Conclusions:**

In Friuli Venezia Giulia, injurious falls are a complication of diabetes relevant from the public health viewpoint. Efforts are needed to screen diabetic patients, review their prescriptions, provide appropriate care, and implement targeted interventions to minimize the individual risk of falls.

## Introduction

Unintentional falls, and fall-related injuries, are associated with a large health and financial burden, and with long-term reduced quality of life.^1,2^ For a decade, falls have been recognized as a relevant issue among diabetic patients, particularly in the elderly. ^3^ Diabetics are generally considered at high risk of falls because of peripheral neuropathy and consequent poor balance. ^4^ Diabetes mellitus; however, is an independent risk factor for falls among older adults, even controlling for poor balance.^5,6^

Various factors have been identified as possibly increasing such risk. Diabetic bones appear to be less strong than non-diabetic bones, for a given bone mass density, possibly because of the accumulation of advanced glycation end products in bone collagen.^7^ In addition, diabetes medications in the thiazolidinediones class are associated with osteoporosis. ^7^ The importance of selecting the most appropriate diabetes medications in elderly patients was also highlighted in a review illustrating that insulin increases the risk of falls and thiazolidinediones the risk of fracture. ^8^ Not only should the choice of antidiabetic medications be considered in the light of this possible danger, but drug prescriptions in general deserves attention. In fact, polypharmacy is another important independent risk factor for falls. ^3,9^ Cognitive impairment was also identified as a risk factor.^3^ Finally, but not less important, age is strongly associated with falls: a combination of old age and diabetes increases the risk of falling 17-fold.^9^

In Italy, no study has been conducted so far to assess the association between diabetes mellitus and accidental falls. In the Friuli Venezia Giulia (FVG) region, located in the Northeast of the country, the literature on the epidemiology of diabetes mellitus, its complications, and management is limited^10-13^ and falls among diabetics have never been studied before. However, the regional health information system of FVG, including several administrative databases which can be integrated with one another, is suitable to explore that issue in an efficient way. Thus, I conducted a research to assess the frequency of falls among the diabetic adult population of FVG, and to identify risk factors, taking advantage of the availability of health-related administrative data.

## Methods

This was a population-based retrospective cohort study conducted in the FVG region, Italy, approximately 1,200,000 inhabitants. I used the regional health information system as the source of information. This system covers the entire regional population and includes various electronic health administrative databases, including the hospital discharge database, the pharmaceutical prescription database, the database of exemptions from medical charges, the Emergency Room (ER) database, and the mortality database. All the databases are linkable at the individual level through an encrypted unique identifier. From those databases, I identified a cohort of subjects with diabetes mellitus who were resident in the region and who were ≥18 years old on December 31, 2014. To avoid loss of cases, I decided to keep in the cohort subjects who died in 2015. However, a sensitivity analysis was conducted excluding those subjects. 

A person was considered diabetic if, before December 31, 2014, he or she had a) a hospital admission with a principal or secondary discharge diagnosis of diabetes mellitus (ICD-9-CM 250), or b) the prescription of at least 3 packages of antidiabetic medications (ATC codes A10Axxx or A10Bxxx) in a 365-day period, or c) an exemption from medical charges because of diabetes. In Italy, potential healthcare beneficiaries may be entitled, because of low income, age, or chronic diseases, to receive free medications and outpatient specialist care. The Italian Ministry of Health assigned codes to all the diseases which entitle patients to exemptions. Currently, they include 56 chronic and disabling diseases, ^14^ including diabetes mellitus, and 47 groups of rare diseases. ^15^ The choice of 3 packages of antidiabetic drugs as a criterion for identifying patients with diabetes, made in agreement with the diabetologists of the regional hospitals: on one side, it reduced false positives due to possible sporadic prescription errors or occasional prescriptions for causes other than diabetes mellitus; on the other side, it was sufficient to capture patients starting chronic drug treatment. 

From the pool of subjects identified as diabetic, I excluded cases of gestational diabetes, who were identified as female subjects who had fulfilled one of the above-listed criteria during the 6 months preceding an obstetric event (i.e., an admission to the hospital with a principal discharge diagnosis with ICD-9-CM code 640-669) and who were prescribed at least 2 packages of blood glucose test strips (ATC code 7AB1B01) during the 3 months preceding the obstetric event. 

For each subject in the cohort, I investigated the accesses to the regional ERs in 2015 and abstracted data on accidental falls from the ER database. The differences between subjects who had at least one fall requiring a visit to an ER and the others were assessed though the chi-square test. 

Of all the falls requiring a visit to an ER, I described the characteristics of the subjects (sex and age class) and of the ER episode (triage color code, outcome: discharged vs admitted to hospital, diagnosis of hip fracture, diagnosis of wrist fracture). 

I also investigated the factors associated with an increased risk of falling among diabetic subjects, through a multivariate logistic regression model including sex, age class (18-44, 45-64. 65-84, ≥85 years), type of last antidiabetic medication prescribed in 2014, if any (insulin, Anatomical Therapeutic Chemical (ATC) code A10Axxx; biguanides, ATC code A10BAxx; sulfonylureas, ATC code A10BBxx; combinations of oral blood glucose lowering blood, ATC code A10BDxx; alpha glucosidase inhibitors, ATC code A10BFxx; thiazolidinediones, ATC code A10BGxx; dipeptidyl peptidase 4 (DPP-4) inhibitors, ATC code A10BHxx; and others, ATC codes A10BCxx and A10BXxx), prescription of medications other than antidiabetic drugs in 2014 (number of active principles and ATC main group: A - Alimentary tract and metabolism, B - Blood and blood forming organs, C - Cardiovascular system, D – Dermatologicals, G - Genito urinary system and sex hormones, H - Systemic hormonal preparations, excl. sex hormones and insulins, J - Antiinfectives for systemic use, L - Antineoplastic and immunomodulating agents, M - Musculo-skeletal system, N - Nervous system, P - Antiparasitic products, insecticides and repellents, R - Respiratory system, S - Sensory organs, V – Various), most recent value of glycated hemoglobin (HbA1c) measured in 2014, if any (not measured, measured in a private laboratory and value not available, >6.5, 6.5-7.4, 7.5-8.4, 8.5-9.4, 9.5-10.4, ≥10.5%), duration of diabetes (0-5, 6-15, and ≥16 years). I also built alternative models to test the possible effect of the prescription of selected types of drugs which are associated to falls in diabetics according to the literature, e.g., the selective serotonin reuptake inhibitors (SSRI, ATC code N06ABxx) .^6^ Results were expressed through the odds ratio (OR) and 95% Confidence Intervals (95%CI). 

All results were considered statistically significant at p-values>0.05. Data analyses were done using SAS 9.2 (SAS Institute Inc., Cary, NC, USA).

All procedures followed were in accordance with the ethical standards of the responsible committee on human experimentation (institutional and national) and with the Helsinki Declaration of 1975, as revised in 2008. Since the study was based only on administrative data without any personal identifier, no informed consent from patients was required.

## Results

On December 31, 2014, there were 80,162 diabetics ≥18 years of age in the FVG region. Of them, 21,562 had at least one access to the regional ERs in the following year. Overall, they accounted for 35,991 accesses. 2967 patients (3.7% of the whole cohort) had at least one access because of an accidental fall: 2739 people had one fall, 200 had 2, 22 had 3, 4 had 4, and 2 had 5 or more, for a total of 3238 events. 

Of all the cohort subjects, only 106 died in 2015 and 12 of them had a fall. 88% of deaths occurred in November and December and the earliest death occurred on March 28th, thus all the cohort subjects had at least 3 months of follow-up and less than 0.02% had less than 11 months of follow-up. Of patients who died in November and December, the cause of death was cancer in 25 cases, heart disease in 24, stroke in 8, bronchitis and/or pneumonia in 7, diabetes mellitus in 5, kidney or urinary disease in 5, disease of the digestive organs in 4, sepsis in 2. For the remaining, the registered cause of death was not specific.

The characteristics of the cohort stratified by fall occurrence (at least one fall in 2015 vs. none) are illustrated in Table 1. The frequency of falls requiring an ER access was higher among females, the elderly, patients treated with thiazolidinediones, DPP-4 inhibitors, sulfonylureas, insulin, those with high levels of HbA1c, longer diabetes duration, and more prescriptions of different active principles in 2014, than among the others. The frequency of falls was also significantly higher among subjects who had been prescribed medicines in the ATC main groups A - Alimentary tract and metabolism (4.7%), B - Blood and blood forming organs (4.7%), C - Cardiovascular system (4.0%), H - Systemic hormonal preparations (4.3%), J - Antiinfectives for systemic use (4.3%), L - Antineoplastic and immunomodulating agents (5.9%), M - Musculo-skeletal system (4.1%), N - Nervous system (5.5%), R - Respiratory system (4.3%), S - Sensory organs (4.4%) in 2014.

**Table 1:** Characteristics of 80,162 diabetics ≥18 years of age resident in the Italian region Friuli Venezia Giulia by outcome (at least one fall requiring a visit to an Emergency Room in 2015 vs none).

	No falls in 2015(N=77,195)		At least one fall in 2015 (N=2967)		p-value
	N	Row %	N	Row %	
**Sex**					>0.0001
Female	35792	95.4	1727	4.6	
Male	41403	97.1	1240	2.9	
**Age class (years)**					>0.0001
18-44	6066	98.6	86	1.4	
45-64	20844	97.7	481	2.3	
65-84	43258	96.1	1775	3.9	
≥85	7027	91.8	625	8.2	
**Last antidiabetic drug in 2014**					>0.0001
None	20457	96.7	702	3.3	
Insulin	7882	95.8	344	4.2	
Biguanides	30445	96.5	1115	3.5	
Sulfonylureas	6529	95.7	294	4.3	
Combinations of oral blood glucose lowering blood	7114	96.2	282	3.8	
Alpha glucosidase inhibitors	499	96.5	18	3.5	
Thiazolidinediones	858	95.1	44	4.9	
DPP-4 inhibitors	668	95.4	32	4.6	
Others	2743	95.3	136	4.7	
**Last HbA1c in 2014**					<0.0001
Not prescribed	27668	96.7	953	3.3	
Prescribed – value unknown	15285	96.6	536	3.4	
<6.5%	10908	96.0	459	4.0	
6.5-7.4%	12997	96.0	546	4.0	
7.5-8.4%	6226	95.9	268	4.1	
8.5-9.4%	2366	95.1	121	4.9	
9.5-10.4%	958	95.3	47	4.7	
≥10.5%	787	95.5	37	4.5	
Diabetes duration (years)					<0.0001
0-5	27710	96.8	917	3.2	
6-15	29109	96.7	1006	3.3	
≥16	20376	95.1	1044	4.9	
Number of active principles other than antidiabetic medications prescribed in 2014					<0.0001
0	6116	98.2	114	1.8	
1-4	26104	97.4	685	2.6	
5-9	29568	95.9	1254	4.1	
≥10	15407	94.4	914	5.6	

Of all the fall events that required an access to an ER in 2015, 22.1% had a white triage tag (minor injury, inappropriate access to ER), 63.7% had a green tag (delayed care), 14.0% had yellow tags (urgent care) and 0.2% had red tags (immediate care is needed). 52.9% of the falls occurred at home; 20.5% required hospital admission. In 258 cases (7.8%) the ER discharge diagnosis was hip fracture, in 9 cases the diagnosis was wrist fracture. Of the 663 patients requiring hospitalization, 406 had a fracture and in 256 it was a hip or writs fracture. Table 2 shows the characteristics of all the fall events by age class. The proportion of subjects with green and yellow tags increased with age, as were the proportion of falls that occurred at home or in residential institutions, the proportion of events requiring admission to the hospital, and the proportion of hip fractures. Only in 5 cases hypoglycemia was mentioned in the ER file.

**Table 2:** Characteristics of 3238 falls requiring access to an Emergency Room in 2015 among diabetics ≥18 years of age resident in the Italian region Friuli Venezia Giulia, by age class.

	18-44 years (N=90)		45-64 years (N=505)		65-84 years (N=1945)		≥85 years (N=698)		p-value
	N	Column %	N	Column %	N	Column %	N	Column %	
**Triage tag**									<0.0001
White	42	46.7	167	33.1	429	22.1	78	11.2	
Green	44	48.9	274	54.3	1234	63.4	510	73.1	
Yellow	4	4.4	63	12.5	277	14.2	109	15.6	
Red	0	0	1	0.2	5	0.3	1	0.1	
**Place of occurrence**									<0.0001
House	30	33.3	203	40.2	1055	54.2	425	60.9	
Residential institution	0	0	18	3.6	89	4.6	72	10.3	
Other	60	66.7	284	56.2	801	41.2	201	28.8	
**Outcome**									<0.0001
Discharged	87	96.7	446	88.3	1565	80.5	477	68.3	
Admitted to hospital	3	3.3	59	11.7	380	19.5	221	31.7	
**Hip fracture**									<0.0001
No	90	100.0	495	98.0	1800	92.5	595	85.2	
Yes	0	0	10	2.0	145	7.5	103	14.8	
**Wrist fracture**									0.6933
No	90	100.0	503	99.6	1941	99.8	695	99.6	
Yes	0	0	2	0.4	4	0.2	3	0.4	

Table 3 describes association of demographic characteristics, medicine prescriptions, glycemic control and the risk of falling among diabetic subjects. After adjusting for the potentially confounding effect of all the other variables in the model, female sex, older age, prescription of thiaziolidinediones, diabetes duration, number of active principles prescribed in 2014, and prescription of medicines regarding the alimentary tract and metabolism (other than antidiabetic drugs), the blood, antibiotics, antineoplastics, and medicines regarding the nervous system were significantly associated with an increased risk of fall. Insulin prescription was associated with a borderline significant 13% increased risk. On the other hand, the prescription of medicines regarding the cardiovascular system was associated with a decreased risk of falls. Although no significant association was observed between falls and HbA1c, the ORs for the categories ≥8.5% suggest the possibility of an increased risk for subjects with poor glycemic control.

**Table 3:** Results of the multivariate logistic regression model assessing the association between demographic characteristics of the subjects, medicine prescript tions and glycemic control in 2014, and the risk of falling in 2015.

	Crude OR	95%CI	Adjusted OR	95%CI
**Sex**				
Male	1.00	-	1.00	-
Female	1.61	1.49-1.73	1.40	1.29-1.52
**Age class (years)**				
18-44	1.00	-	1.00	-
45-64	1.63	1.29-2.05	1.62	1.27-2.05
65-84	2.89	2.33-3.60	2.40	1.89-3.04
≥85	6.27	4.99-7.88	4.60	3.60-5.88
**Last antidiabetic drug in 2014**				
None	0.99	0.85-1.03	1.03	0.92-1.15
Insulin	1.19	1.05-1.35	1.02	0.89-1.17
Biguanides	1.00	-	1.00	-
Sulfonylureas	1.23	1.08-1.40	1.05	0.92-1.20
Combinations of oral blood glucose lowering blood	1.08	0.95-1.24	0.98	0.86-1.13
Alpha glucosidase inhibitors	0.98	0.61-1.58	0.88	0.54-1.41
Thiazolidinediones	1.40	1.03-1.91	1.37	1.00-1.87
DPP-4 inhibitors	1.31	0.91-1.87	1.13	0.79-1.63
Others	1.35	1.13-1.62	1.01	0.84-1.22
**Last HbA1c in 2014**				
Not prescribed	0.82	0.74-0.91	0.95	0.84-1.06
Prescribed – value unknown	0.83	0.74-0.94	0.91	0.81-1.03
<6.5%	1.00	0.88-1.14	1.05	0.92-1.19
6.5-7.4%	1.00	-	1.00	-
7.5-8.4%	1.02	0.88-1.19	0.97	0.83-1.13
8.5-9.4%	1.22	0.99-1.49	1.13	0.92-1.39
9.5-10.4%	1.17	0.86-1.58	1.17	0.85-1.59
≥10.5%	1.12	0.80-1.57	1.22	0.87-1.73
**Diabetes duration (years)**				
0-5	1.00	-	1.00	-
6-15	1.04	0.95-1.44	1.01	0.92-1.11
≥16	1.55	1.41-1.69	1.27	1.15-1.40
Number of active principles other than antidiabetic medications prescribed in 2014				
0	0.71	0.58-0.87	0.75	0.60-0.95
1-4	1.00	-	1.00	-
5-9	1.62	1.47-1.77	1.18	1.04-1.34
≥10	2.26	2.04-2.50	1.32	1.11-1.57
**ATC main groups prescribed in 2015 (yes vs no)**				
A - Alimentary tract and metabolism	1.70	1.58-1.84	1.12	1.02-1.22
B - Blood and blood forming organs	1.76	1.63-1.90	1.16	1.05-1.28
C - Cardiovascular system	1.52	1.38-1.68	0.80	0.70-0.91
D – Dermatologicals	1.04	0.83-1.30	0.95	0.76-1.19
G - Genito urinary system and sex hormones	1.23	1.12-1.35	0.95	0.83-1.09
H - Systemic hormonal preparations	1.32	1.23-1.42	0.97	0.88-1.07
J - Antiinfectives for systemic use	1.72	1.46-2.08	1.10	1.01-1.19
L - Antineoplastic and immunomodulating agents	0.94	0.86-1.02	1.34	1.13-1.59
M - Musculo-skeletal system	1.38	1.27-1.50	0.94	0.86-1.02
N - Nervous system	1.03	0.76-1.41	1.38	1.27-1.50
P - Antiparasitic products, insecticides and repellents	0.98	0.89-1.08	1.03	0.76-1.41
R - Respiratory system	0.92	0.81-1.06	0.98	0.89-1.08
S - Sensory organs	0.92	0.81-1.06	0.92	0.81-1.06
V – Various	1.10	0.82-1.48	1.10	0.82-1.48

SSRIs were prescribed to 5372 patients in 2014: 13.3% of patients with a fall and 6.4% of the others. In the model including SSRIs instead of the ATC main groups, prescription of these antidepressants was associated with a 70% increase in risk (OR=1.70, 95%CI: 1.52-1.90).

In the sensitivity analyses conducted after excluding patients who died in 2015 the results did not change (data not shown).

## Discussion

In Friuli Venezia Giulia, diabetics had a 3.7% one-year risk of falls requiring ER visit, much lower than the risk reported in a small study conducted in the UK, where 39% of the subjects enrolled reported a fall in the last 12 months.^16^ My study, however, considered all the adult diabetic population, was based on administrative data, and examined only the events that required ER visit. On the other hand, the English study was restricted to the elderly, who are at increased risk, was based on self-reports, which are prone to bias, and included any fall, regardless of severity. In another study among 362 Korean diabetic subjects ≥50 years of age, 10% of males and 21% of females experienced a fall during the previous year. ^17^ As in the English study, falls were self-reported and the study was not restricted to falls requiring ER visit, thus the results are not directly comparable with mine. 

In my population, the risk of falling differed across age categories: among subjects ≥85 years of age, it was 8.2%, i.e., 2 times higher than in the age group 65-84 and 4 times higher than in the group 45-64, confirming the strong role of age as a risk factor.^9^ In addition, the outcomes were more serious among the elderly, who were admitted to the hospital and had hip fractures much more frequently than the younger subjects. Among patients ≥85 years of age, 10% of the falls occurred in residential institutions, which should provide protected living environments. In those institutions, guidelines for appropriate drug prescription and administration and structured diabetes care are warranted. In FVG, where regional guidelines for the integrated care of diabetic patients have been released in 2015.^18^ the importance of evaluating the prescriptions of medicines other than antidiabetics should be emphasized. In fact, in my study, the risk of falls increased with the number of different active principles prescribed in the previous year. This highlights the importance of evaluating comorbidities in diabetics and draws attention on the dangers of polypharmacy in these patients.^9^

In addition to the number of active principles, I observed an increased risk of falls among patients with prescriptions of medicines in the ATC main groups A - Alimentary tract and metabolism, B - Blood and blood forming, J - Antiinfectives for systemic use, L - Antineoplastic and immunomodulating agents, N - Nervous system. It is unclear, however, whether the increased risk is associated with the medicines themselves, or with the diseases for which the medicines were indicated. The second hypothesis (i.e., the drug is a marker for the underlying disease) seems to be supported by the increased risk I observed among subjects with prescriptions of SSRIs, a result which is not consistent with findings by others.^6^

Peripheral neuropathy, which may determine balance impairments4 and hypoglycemia ^19^ are other known risk factors for falls among diabetics. In diabetic patients with neuropathy, lower extremities nerve decompression appeared to improve nerve conduction, restore lower limb feeling and motor function, ^20^ although a recent controlled trial in patients with painful diabetic polyneuropathy showed no evidence that surgical decompression of nerves influences stability. ^21^ In my study, however, both peripheral neuropathy and hypoglycemia were only mentioned in 5 cases in the ER records. Awareness of the importance of recording information on those factors is being heightened among the personnel of the regional ERs. In the study by Kachroo et al., ^19^ patients who experienced hypoglycemia were more commonly prescribed sulfonylurea, insulin, and thiaziolidinedions than the others. In my study, only prescription of thiaziolidinedions was associated with increased risk of falling, however, I do not know whether the excess risk is due to hypoglycemia, bone effects, ^7^ or weight gain. Unfortunately, the regional health information system of FVG does not contain information on weight or BMI of the population and I cannot assess its role in facilitating falls. 

Although not significantly, falls occurred more frequently in patients with poorer glycemic control. In my population, not only a large proportion of diabetics had poor glycemic control, but more than one third of patients was not prescribed any HbA1c measurement during 2014. The regional Health Authority of FVG must closely monitor the implementation of the guidelines, ^18^identify the general practitioners who do not provide adequate care to their patients, and discuss with them the challenges they face and possible strategies to achieve the proposed goals in terms of glycemic control.

A number of other interventions that might reduce the risk of falls in elderly diabetic patients are described in the literature, from balance training ^22^ to newer strategies such as tactile intervention ^23^ or whole-body vibration training. ^24^

This study was made possible by the availability of a health information system with coverage of the entire population of the FVG region. The use of administrative data made this research efficient and free from self-reporting bias. However, there may be some limitations. First, the validity of findings depends on the completeness of data recording and on the quality of coding. In addition, the algorithms used to identify the cohort of diabetic patients might misclassify some subjects: although they were agreed upon by a panel of regional health professionals very experienced in the management of patients with diabetes, sensitivity and specificity could not be formally tested since a true gold standard is not available in FVG. 

Despite these potential limitations, this research shows that diabetes affects a large proportion of the population in FVG and injurious falls are a relevant complication of the disease, although often neglected. In this region, careful screening of the diabetic population and targeted strategies to minimize the individual risk factors for falls, involving general practitioners, specialists, public health practitioners, patients and their families, are encouraged.
